# Validation of the Leiden Inventory for the Child’s Well-Being in Daycare (LICW-D) Questionnaire in Norwegian Early Childhood Education and Care Centers

**DOI:** 10.3389/fpsyg.2021.767137

**Published:** 2021-11-25

**Authors:** Catharina P. J. van Trijp, Ratib Lekhal, May Britt Drugli, Veslemøy Rydland, Elisabet Solheim Buøen

**Affiliations:** ^1^Department of Communication and Culture, BI Norwegian Business School, Oslo, Norway; ^2^Department of Education, University of Oslo, Oslo, Norway; ^3^Regional Centre for Child and Adolescent Mental Health – Central Norway, Norwegian University of Science and Technology, Trondheim, Norway; ^4^Centre for Studies of Educational Practice, Inland Norway University of Applied Sciences, Elverum, Norway; ^5^Eastern and Southern Norway, Regional Center for Child and Adolescent Mental Health, Oslo, Norway

**Keywords:** well-being, toddlers, ECEC, professional caregivers, Norway, confirmatory factor analysis

## Abstract

The promotion of children’s development and well-being is a core concept in Early Childhood Education and Care (ECEC) quality frameworks. Yet, few validated instruments measuring young children’s well-being exist. This study examined the validity of The Leiden Inventory for the Child’s Well-being in Daycare (LICW-D) (De Schipper et al., 2004b) in a sample of toddlers (*n* = 1,472) attending ECEC centers in Norway, using confirmatory factor analysis. Factorial invariance across gender and concurrent validity were also investigated. Indicators of concurrent validity were problem behaviors and difficult temperament, as rated by professional caregivers. Results showed a marginally acceptable fit for the hypothesized one-factor model, when allowing the measurement error of four item pairs to be correlated. This slightly modified model showed satisfactory concurrent validity, and factorial invariance across gender was confirmed.

## Introduction

There is an increasing international focus on the promotion, measurement, and monitoring of people’s well-being (Ben-Arieh, 2008; Huppert and So, 2013). This focus seems to be encouraged by the findings of multiple international studies showing positive consequences of a high level of social-emotional well-being (“well-being”) on health, learning, productivity, social relationships, and life expectancy (Lyubomirsky et al., 2005; Huppert, 2009). For example, children with a strong sense of well-being engage more confidently and positively with their learning environment. This might help them to profit more fully from the education and care settings wherein they participate (Department for Education and Child Development, 2016). Moreover, it may support children’s development and experience of quality of life (Mashford-Scott et al., 2012). A strong sense of well-being seems to be particularly important during the early years of life. Neurobiological studies showed that there is a peak in the neuroplasticity of the brain during the first years of life (Blakemore and Frith, 2005). During this period, the child is more sensitive to the level of support from their environment, which determines how the foundations for well-being and learning develop in the brain (National Scientific Council on the Developing Child, 2007). Thus, children’s well-being, development, and later life outcomes are largely dependent on children’s experiences with their environment. Monitoring children’s well-being as early as possible might prevent certain developmental challenges in children’s current and later life. However, at present there is a lack of well-validated instruments that measures children’s well-being during their first years of life.

In many European countries, besides the home environment, most children spend considerable time in Early Childhood Education and Care (ECEC) during their early years (Council of the European Union, 2019). In Norway, where the present study was conducted, 85.4% of 1- to 2-year-olds attended an ECEC center in 2020 (Statistics Norway, 2021). Moreover, Norway is known for its holistic approach to ECEC, which means that ECEC centers aim to promote children’s well-being, learning, and development, for all ages and with similar ECEC quality, before the children enter compulsory schooling (Organization for Economic Co-operation and Development [OECD], 2015). This focus on the promotion of young children’s well-being and development is also present in international ECEC quality frameworks and guidelines (Organization for Economic Co-operation and Development [OECD], 2012; Sylva et al., 2015; Council of the European Union, 2019). The considerable attention to children’s well-being is promising, as it seems that well-being during the early years and children’s experience in ECEC form a foundation for their current and later life outcomes. However, when it comes to research on children’s well-being in ECEC, there are some limitations.

At present, there is a lack of well-validated instruments that measure children’s well-being in ECEC. Zachrisson and Lekhal (2014) found that only a few studies have examined the impact of ECEC on children’s well-being directly. De Schipper et al. (2003, 2004a,b) measured children’s well-being directly by the degree to which a child feels at ease with his or her caregivers, and how comfortable the child is with other children in the group and in the physical setting of the center. Instead, most studies focus on the impact of ECEC on social-emotional development, behavior, and cognitive and academic achievements, which can be considered as proxies for children’s well-being (Zachrisson and Lekhal, 2014). Other examples of proxies that have been used to study children’s well-being are children’s health and safety, material resources, education, quality of school life, personal relationships, risk behavior, and housing and environment (Bradshaw and Richardson, 2009; Organization for Economic Co-operation and Development [OECD], 2009). Zachrisson and Lekhal (2014) stressed the need for studies that measure the impact of ECEC on children’s well-being. However, the use of proxies by multiple studies to measure well-being underlines the urgent need for an instrument that measures children’s well-being in ECEC directly.

One of the few available instruments that measure young children’s well-being in ECEC is the Leiden Inventory for the Child’s Well-being in Daycare (LICW-D) (De Schipper et al., 2004b). To our knowledge, this questionnaire has only been validated in samples in the Netherlands. There is therefore a need to validate this instrument in other samples and countries as well. For this reason, the present study investigated the psychometric properties of the LICW-D in a large sample of toddlers attending ECEC centers in Norway. A well-validated instrument will provide national and international research, policy, and practice with a tool for measuring children’s well-being in ECEC.

The LICW-D is an elaboration of the Well-being Scale of Van IJzendoorn et al. (1998). The latter focused on the degree to which a child feels at ease in the professional child-care setting. De Schipper et al. (2004b) extended the inventory by focusing on well-being as the degree to which a child feels at ease with his or her caregivers, and how comfortable the child is with other children in the group and in the physical setting of the center. The first version of the LICW-D consisted of 28 items and was developed to identify four factors related to well-being: general well-being, and well-being with group members, in the presence of caregivers, and within the physical environment. Professional caregivers in daycare centers responded to the items on a 6-point Likert scale ranging from 1 (*never*) to 6 (*always*) (De Schipper et al., 2004b).

De Schipper et al. (2004b) validated the LICW-D with 186 professional caregivers of 186 children, aged 6–30 months, enrolled in 113 different daycare centers in the Netherlands. The intended four-factor structure was not confirmed. In particular, items that were related to the child feeling at ease did not show a clear pattern. In their further analysis, De Schipper et al. (2004b) used a one principal component approach that included 12 items that correlated significantly with the main item “This child enjoys attending the day care center.” A one factor solution fitted the data most adequately. The average component loading for this analysis was 0.55 (ranging from 0.33 to 0.69), and the internal consistency was good: Cronbach’s alpha 0.81 (*n* = 159). Thus, the final model showed a one factor, 12-item questionnaire.

The few studies in the Netherlands that have used the LICW-D found correlations with different child characteristics. Children with a more difficult temperament (e.g., showing more irritable distress and more difficulty to adapt to novelty) had a lower feeling of well-being in ECEC (De Schipper et al., 2003, 2004a). A lower feeling of well-being also correlated with more internalizing (De Schipper et al., 2004b; Gevers Deynoot-Schaub and Riksen-Walraven, 2006) and externalizing behavior problems (Gevers Deynoot-Schaub and Riksen-Walraven, 2006). Children with an easier temperament showed more well-being and less internalizing and total behavior problems (De Schipper et al., 2004a). Gender differences were not found (De Schipper et al., 2003).

This study examined the factor structure of the 12-item LICW-D in a large sample of 1- to 3-year-olds in ECEC centers (center-based daycare) in Norway using confirmatory factor analysis (CFA). In addition, we investigated the concurrent validity of the instrument and whether there is factorial invariance across gender. The aim was to validate the LICW-D in a Norwegian toddler sample. As a result, this study might provide national and international research, policy, and practice with a tool for measuring children’s well-being in ECEC and for developing systematic knowledge.

We hypothesized that the one-factor model suggested by De Schipper et al. (2004b) would be supported and that there would be no differences between boys and girls (De Schipper et al., 2003). In line with earlier studies, we also hypothesized that children scoring high on well-being would be more likely characterized by a less difficult temperament (De Schipper et al., 2003, 2004a), and show fewer symptoms of internalizing, externalizing, and total behavior problems (De Schipper et al., 2004b; Gevers Deynoot-Schaub and Riksen-Walraven, 2006).

## Materials and Methods

### Procedures

The present study is part of the larger Thrive by 3 cluster randomized controlled trial study (Trygg før 3) (Lekhal et al., 2020). Thrive by 3 is a model of intervention and implementation of quality building and control in Norwegian ECEC centers to strengthen 1- to 3-year-olds’ mental health, social and cognitive development, and well-being and to reduce their cortisol levels (stress). All data was collected through electronic questionnaires filled out by parents and professional caregivers, and through observations of the staff-child interactions in ECEC centers. Seven municipalities/city districts were invited and consented to participate in the study – four in Eastern Norway and three in Central Norway. The managers of the ECEC centers received an e-mail (or letter if needed) with an electronic link to the written informed consent form to decide on the ECEC center’s participation and their own participation. In addition, the managers forwarded the e-mail with the written informed consent from the Thrive by 3 study to all professional caregivers, parents, and children at the center. A total of 187 units/groups in 78 ECEC centers agreed to participate. The staff-child ratio in each unit/group was at least one professional caregiver working with three children. Parents provided written consent for their child. The study was approved by the Regional Committees for Medical and Health Research Ethics South East Norway and by the Norwegian Centre for Research Data.

### Participants

The present study used the T1 data (baseline data) from the electronic questionnaires that were filled out by the professional caregivers who had the closest relationship with the child. A total of 1,472 children (746 boys, 726 girls) aged 7 months to 37 months (*M* = 21.4 months, *SD* = 6.1) who were part of one of the 184 units/groups in 78 ECEC centers, were answered by a professional caregiver on the LICW-D.

### Measures

#### Well-Being

The 12 items of the LICW-D (De Schipper et al., 2004b) were used to measure children’s well-being. The LICW-D was translated from English to Norwegian and then translated back from Norwegian to English. In Norwegian, the distinction between the answer categories “regularly” and “often” was not clear. Therefore, a 5-point Likert-scale was used in the present study: 1 (*never*), 2 (*seldom*), 3 (*sometimes*), 4 (*often*), 5 (*always*), instead of the 6-point Likert-scale that was proposed by De Schipper et al. (2004b).

#### Indicators of Concurrent Validity

##### Difficult temperament

Two scales, frustration and soothability, from The Early Childhood Behavior Questionnaire (ECBQ) short version (Putnam et al., 2010) were used to assess children’s difficult temperament. These scales are part of the larger negative affect factor in the ECBQ short version. Frustration was assessed by six items that focused on negative affect related to interruption of ongoing tasks or goal blocking. Soothability was assessed by five items that focused on the rate of recovery from peak distress, excitement, or general arousal. Questions were answered on a 7-point Likert-scale ranging from 1 (*never*) to 7 (*always*) in addition to *does not apply*. The internal consistency ranged from acceptable to good with a Cronbach’s alpha of 0.70 for the soothability scale, and 0.88 for the frustration scale.

##### Problem behavior

The Child Behavior Checklist Teacher Report Form for Ages 1.5–5 (CBCL-TRF/11/2-5) (Achenbach and Rescorla, 2000) was used to measure internalizing, externalizing, and total behavior problems of the children. Internalizing problems were assessed by a total of 36 items divided over the four subscales: emotionally reactive, anxious/depressed, somatic complaints, and withdrawn. Externalizing problems were assessed by a total of 24 items divided over the two subscales: attention problems and aggressive behavior. For the total problems (99 items), the scales sleep problems and other problems are assessed in addition to the internalizing and externalizing problems. The professional caregivers responded to the items on a scale from 0 (*not true*) to 2 (*very true or often true*). The internal consistency ranged from good to excellent with a Cronbach’s alpha of 0.88 for the internalizing, 0.90 for the externalizing, and 0.95 for the total behavior problems scale.

### Analysis

To examine the factorial validity and gender invariance of the LICW-D, we conducted a CFA, multigroup CFA, and CFA with a covariate (MIMIC; multiple indicators, multiple causes). Alternative models were explored by exploratory factor analysis (EFA). The concurrent validity of the LICW-D was investigated by means of bivariate correlations. All analyses were conducted with Mplus Version 8 (Muthén and Muthén, 2017).

#### Factor Structure

Initial data diagnostics showed that the observed responses on the LICW-D were discrete realizations of a limited number of categories on most items. Thus, the assumption of continuity was broken, and data was handled as categorical by using a weighted least square estimator (WLSMV) (Flora and Curran, 2004; Nussbeck et al., 2006). The one-factor model, and alternative models were evaluated by using four commonly reported indices: comparative fit index (CFI), Tucker-Lewis index (TLI), root mean square error of approximation (RMSEA), and standardized root mean square residual (SRMR). Since the Chi-square is highly sensitive to sample size, trivial discrepancies can lead to the rejection of a highly satisfactory model (Brown, 2015). Therefore, this statistic should be interpreted with caution when examining the overall fit of the LICW-D. Good model fit was defined as CFI > 0.95, TLI > 0.95, RMSEA ≤ 0.05, and SRMR ≤ 0.05, and acceptable model fit was defined as CFI and TLI 0.90 – 0.95, RMSEA 0.06 – 0.10, and SRMR 0.06 – 0.08 (e.g., MacCallum et al., 1996; Hu and Bentler, 1999). To evaluate factor loadings of each item we used the *R*^2^ estimates (≥0.25) and standardized factor loadings (≥0.40); a low *R*^2^ indicates a high level of error for an item (Brown, 2015). Factor loadings of 0.32 were rated as poor, 0.45 as fair, 0.55 as good, 0.63 as very good, and 0.71 and above as excellent (Comrey and Lee, 1992). A low *R*^2^ indicates a high level of error for an item (Brown, 2015). For the CFA of the one-factor solution we also examined the modification indices (MI) above 10 coupled with high-expected parameter change (EPC ≥ 0.40). A large modification index indicates that removing the equality constraint or freeing the parameter could result in a better model fit (Muthén and Muthén, 2017). To identify acceptable EFA solutions we used the following criteria: Each factor should have an eigen-value of 1 or above (Kline, 2016), each factor should be significantly loaded by a minimum of three variables, each variable should not load significantly on multiple factors, the internal consistency of each factor should be ≥ 0.70, and all factors should be theoretically meaningful (Fabrigar and Wegener, 2012).

#### Factorial Invariance Across Gender

We studied the factorial invariance across gender by conducting a multigroup CFA. A Chi-square difference test was calculated with the WLSMV estimator, to examine the fit of nested CFA models (Muthén and Muthén, 2017). To further study gender invariance, gender was used as a covariate in an MIMIC analysis.

#### Concurrent Validity

Concurrent validity was examined by bivariate correlations between a child’s well-being, difficult temperament, and behavior problems. Cutoff values for the interpretation of the correlations were tiny (<0.10), small (0.10 – 0.29), medium (0.30 – 0.49), and large (≥0.50) (Field, 2018).

## Results

### Factor Structure

Examining the one-factor model originally proposed by De Schipper et al. (2004b), the CFA indicated a poor model fit on all fit indices, except the SRMR, which was acceptable (χ^2^(54) = 1850.402, *p* < 0.00001, CFI = 0.88, TLI = 0.86, RMSEA = 0.15, SRMR = 0.07). A closer look at the parameter estimates showed that the standardized factor loadings ranged from 0.59 to 0.79 and the *R*^2^ from 0.35 to 0.62. Item 7 (“This child has difficulty saying goodbye to the parent, he/she is distressed or inconsolable”) had the lowest values on the parameter estimates, and item 10 (“This child does not feel at ease in the group”) had the highest. Nevertheless, none of the items violated the cutoff values for standardized factor loadings and *R*^2^.

### Test for Alternative Measurement Model

To discover the cause of the poor model fit, we conducted subsequent CFAs. However, the model fit might be affected by sample-specific variance when single-sample *post hoc* modifications are conducted. Therefore, we split our large sample in two random halves: Sample A (*n* = 748 children) and B (*n* = 724 children). We were thus able to explore modifications of the LICW-D model in one half (Sample A) followed by a cross-validation of the final model in the second half (Sample B) and the whole sample.

Although the initial LICW-D was developed to identify a four-factor structure (De Schipper et al., 2004b), there have been no reports of testing a multifactor solution of the 12-item LICW-D scale. We therefore based our test for alternative measurement models on the originally suggested one-factor solution first.

We started by testing the original model in half of the sample (Sample A). The model fit was poor to acceptable (χ^2^(54) = 864.055, *p* < 0.00001, CFI = 0.91, TLI = 0.89, RMSEA = 0.14, SRMR = 0.06), and similar to the one found in the whole sample. Standardized factor loadings ranged from 0.62 to 0.80 and the *R*^2^ from 0.38 to 0.64. Again, item 7 had the lowest values on the parameter estimates, and item 10 and 4 (“This child trusts all the children at the daycare center”) had the highest. Inspection of the MI as a guide in search of model misspecification indicated that allowing the measurement error of item 7 (“This child has difficulty saying goodbye to the parent, he/she is distressed or inconsolable”) and item 5 (“This child is sometimes reluctant to attend the daycare center”) to be correlated was associated with the largest MI (185.40) and EPC (0.54). We also allowed correlations between the measurement error of item 11 (“This child actively seeks the company of other children”) and item 6 (“This child tends to avoid contacts with other children”) (MI = 104.04, EPC = 0.41); item 12 (“This child really enjoys the games and play material at the daycare center”) and item 11 (MI = 115.64, EPC = 0.48); and item 4 (“This child trusts all the children at the daycare center”) and item 2 (“This child does not feel at ease with some of the children”) (MI = 69.84, EPC = 0.40). Taken together, as Table 1 shows, these four changes resulted in an acceptable fit for Sample A regarding the TLI and RMSEA, and good regarding the CFI and SRMR. Table 1 also shows that this modified measurement model replicated relatively well in Sample B and the whole sample. In Sample B all fit indices were acceptable, except the RMSEA. In the whole sample, the RMSEA was also non-acceptable, but the CFI and TLI were acceptable, and the SRMR was good. Table 2 presents factor loadings and *R*^2^ of the modified models for all three samples. Items 2, 7, and 11 showed the lowest values, but none of the items violated the cutoff values for low factor loadings (<0.40) and very low *R*^2^ estimates (<0.25). The LICW-D showed good internal consistency with a Cronbach’s alpha of 0.87.

**TABLE 1:** Fit indices of modified model for the different samples.

**Sample**	**χ^2^ (*df*)**	**CFI**	**TLI**	**RMSEA**	**SRMR**
Sample A (*n* = 748)	427.792 (50)*	0.96	0.94	0.10	0.05
Sample B (*n* = 724)	551.492 (50)*	0.93	0.90	0.12	0.06
Whole sample (*n* = 1,472)	916.701 (50)*	0.94	0.93	0.11	0.05

χ^2^ = Chi-Square; df = degrees of freedom; CFI, comparative fit index; TLI, Tucker-Lewis Index; RMSEA, root-mean-squared error of approximation; SRMR, standardized root-mean-squared residual, *p < 0.00001.

**TABLE 2:** Standardized factor loadings and *R*^2^ of the modified measurement model for the different samples.

**LICW-D item number and descriptions**	**Sample A (*n* = 748)**	**Sample B (*n* = 724)**	**Whole sample (*n* = 1,472)**
1. This child enjoys attending the daycare center	0.79 (0.63)	0.77 (0.60)	0.78 (0.61)
2R. This child does not feel at ease with some of the children	0.60 (0.36)	0.57 (0.33)	0.59 (0.35)
3. This child is happy to see the professional caregiver(s) when he/she is dropped off	0.73 (0.54)	0.66 (0.44)	0.70 (0.49)
4. This child trusts all the children at the daycare center	0.78 (0.61)	0.72 (0.51)	0.76 (0.57)
5R. This child is sometimes reluctant to attend the daycare center	0.66 (0.43)	0.61 (0.38)	0.63 (0.40)
6R. This child tends to avoid contacts with other children	0.64 (0.41)	0.69 (0.48)	0.66 (0.44)
7R. This child has difficulty saying goodbye to the parent, he/she is distressed or inconsolable	0.56 (0.31)	0.50 (0.25)	0.53 (0.28)
8. This child feels at ease with all the professional caregivers	0.65 (0.43)	0.62 (0.39)	0.64 (0.41)
9R. This child does not feel comfortable outside the playground	0.73 (0.53)	0.69 (0.47)	0.71 (0.50)
10R. This child does not feel at ease in the group	0.82 (0.67)	0.81 (0.65)	0.81 (0.66)
11. This child actively seeks the company of other children	0.60 (0.36)	0.53 (0.28)	0.57 (0.32)
12. This child really enjoys the games and play material at the daycare center	0.66 (0.44)	0.69 (0.48)	0.67 (0.45)

*R = reversed for analyses. All standardized factor loadings were significant at p < 0.001. R^2^ is presented in parentheses.*

To be sure that items 2, 7, and 11 did not cause the poorer model fit, we took out these items one by one to see if the model improved. We removed item 7 because this item had the lowest values on the parameter estimates compared to items 2 and 11. After removing item 7, the model fit (χ^2^(44) = 1405.541, *p* < 0.00001, CFI = 0.90, TLI = 0.88, RMSEA = 0.15, SRMR = 0.06) did not improve compared to the original 12-item one-factor model (χ^2^(54) = 1850.402, *p* < 0.00001, CFI = 0.88, TLI = 0.86, RMSEA = 0.15, SRMR = 0.07). The internal consistency slightly decreased with a Cronbach’s alpha of 0.86. Next, we removed item 11 since this item had the second lowest values on the parameter estimates. Again, the model fit (χ^2^(35) = 990.627, *p* < 0.00001, CFI = 0.93, TLI = 0.90, RMSEA = 0.14, SRMR = 0.06) did not get better compared to the original model, and the internal consistency decreased slightly with a Cronbach’s alpha of 0.85. Finally, we removed item 2, which did not improve the model fit (χ^2^(27) = 641.895, *p* < 0.00001, CFI = 0.94, TLI = 0.93, RMSEA = 0.12, SRMR = 0.05), and the internal consistency became slightly lower with a Cronbach’s alpha of 0.84. We therefore kept all 12 items for the next analyses.

Even though there are no reports of testing a multi-factor solution of the 12-item LICW-D scale, we conducted an EFA to further examine the factor structure of these 12 items, both in Sample A and in the whole sample. First, we conducted an EFA in Sample A. The results showed that only the one-factor solution met all criteria as outlined by Fabrigar and Wegener (2012) and Kline (2016) to identify an acceptable EFA solution. Although multiple factor solutions showed a better model fit, all of them had several items that loaded significantly on multiple factors. In addition, in all solutions with three or more factors, factor three and four had an eigen-value below 1 and/or consisted of only one or two items. Therefore, we continued to explore the two-factor model only. The two-factor model had five items that loaded significantly on both factors. Item 8 was loading almost equally on both factors. We retained these items on the factor with the highest loading. The two-factor model showed that items 2, 4, 6, 8, 9, 10, 11, 12 loaded on one factor and had a good internal consistency with a Cronbach’s alpha of 0.84, which represented the items that are mainly focusing on how comfortable the child feels at the center and in interactions with peers and professional caregivers. Items 1, 3, 5, 7 focus on the child’s well-being during arrival and attendance at the ECEC center and were loading on the other factor (see Table 2 for a description of the items). These items showed good internal consistency with a Cronbach’s alpha of 0.78. Nevertheless, the model fit of the two-factor model (χ^2^(43) = 521.828, *p* < 0.00001, CFI = 0.95, TLI = 0.92, RMSEA = 0.12, SRMR = 0.06) was not better than the model fit of the modified one-factor model (χ^2^(50) = 916.701, *p* < 0.00001, CFI = 0.94, TLI = 0.93, RMSEA = 0.11, SRMR = 0.05). Next, we conducted an EFA in whole sample as well, but we found similar results as in Sample A. The model fit of the two-factor model in the whole sample (χ^2^(43) = 1043.025, *p* < 0.00001, CFI = 0.94, TLI = 0.90, RMSEA = 0.13, SRMR = 0.06) was similar to the two-factor model in Sample A and the modified one-factor model.

To further examine the potential of a two-factor solution, we conducted a two-factor CFA in Sample B. However, the model fit was not better (χ^2^(53) = 626.695, *p* < 0.00001, CFI = 0.92, TLI = 0.90, RMSEA = 0.12, SRMR = 0.06) compared to the two-factor model in Sample A and the modified one-factor model. Considering that the two-factor model did not show a better model fit in any of the samples and had items that loaded significantly on both factors, we decided to continue our analyses with the 12-item modified one-factor model.

### Factorial Invariance Across Gender

Based on previous research, we hypothesized that there would be no differences between boys and girls on the LICW-D. Therefore, the modified LICW-D model was used in a multigroup CFA to study the equivalence of factorial validity across gender. First, we tested the measurement invariance to assess the psychometric equivalence across gender. The model fit indices of the invariance analyses are presented in Table 3. The configural invariance model showed a marginally acceptable fit, because of the high RMSEA. The metric and scalar invariance models both showed an acceptable fit. Since the scalar invariance model was significantly worse than the metric invariance model, we checked for partial scalar invariance by following the procedures mentioned by several studies (Schmitt et al., 2011; Jung and Yoon, 2016; Putnick and Bornstein, 2016). We applied the forward approach by adding item intercept constraints and retesting the model. Constraining individual item intercepts did not significantly worsening the model fit, but the fully constrained model was significantly worse compared to the unconstrained model. Then we applied the backward process by constraining all items and compared the intercepts of boys and girls to identify the items that differed the most between the groups, and sequentially releasing them. The model fit was significantly worse than the unconstrained model, leading us to conclude that there was no partial scalar invariance.

**TABLE 3:** Fit indices for the invariance analyses for the modified model for boys and girls.

	**χ^2^ (*df*)**	**CFI**	**TLI**	**RMSEA**	**90% CI**	**SRMR**	**Δχ^2^ (*df*)**	***p***
Configural invariance	945.271 (100)*	0.95	0.93	0.11	–	0.05	–	–
Metric invariance	819.181 (111)*	0.96	0.95	0.09	0.087, 0.099	0.05	9.003 (11)	0.6216
Scalar invariance	783.989 (142)*	0.96	0.96	0.08	0.073, 0.084	0.05	69.901 (31)	0.0001

χ^2^, Chi-Square; df, degrees of freedom; CFI, comparative fit index; TLI, Tucker-Lewis Index; RMSEA, root-mean-squared error of approximation; CI, confidence interval; SRMR, standardized root-mean-squared residual, *p < 0.00001.

We applied the modified one-factor CFA model to boys (*n* = 746) and girls (*n* = 726) separately to see if the model was acceptable in both groups. Table 4 shows a slightly better model fit for boys than girls, but not significantly. Both models were good on the SRMR and acceptable on all other fit indices, except the RMSEA for girls. In addition, the CFI for boys was good. Therefore, the model fit for boys was considered as acceptable and the model fit for girls as marginally acceptable. The LICW-D showed good internal consistency with a Cronbach’s alpha of 0.86 for boys and 0.87 for girls. Table 5 presents factor loadings and *R*^2^ of the modified models for both boys and girls, which also shows factorial invariance across gender.

**TABLE 4:** Fit indices for the modified model for boys and girls.

**Sample**	**χ^2^ (*df*)**	**CFI**	**TLI**	**RMSEA**	**SRMR**
Boys (*n* = 746)	414.260 (50)*	0.96	0.94	0.10	0.05
Girls (*n* = 726)	532.828 (50)*	0.94	0.92	0.12	0.05

χ^2^, Chi-Square; df, degrees of freedom; CFI, comparative fit index; TLI, Tucker-Lewis Index; RMSEA, root-mean-squared error of approximation; SRMR, standardized root-mean-squared residual, *p < 0.00001.

**TABLE 5:** Standardized factor loadings and *R*^2^ of the modified measurement model for boys and girls.

**LICW-D item number and descriptions**	**Boys (*n* = 746)**	**Girls (*n* = 726)**
1. This child enjoys attending the daycare center	0.81 (0.65)	0.76 (0.58)
2R. This child does not feel at ease with some of the children	0.59 (0.35)	0.59 (0.35)
3. This child is happy to see the professional caregiver(s) when he/she is dropped off	0.70 (0.49)	0.70 (0.49)
4. This child trusts all the children at the daycare center	0.76 (0.57)	0.76 (0.57)
5R. This child is sometimes reluctant to attend the daycare center	0.62 (0.39)	0.66 (0.43)
6R. This child tends to avoid contacts with other children	0.64 (0.41)	0.68 (0.47)
7R. This child has difficulty saying goodbye to the parent, he/she is distressed or inconsolable	0.52 (0.27)	0.55 (0.30)
8. This child feels at ease with all the professional caregivers	0.67 (0.45)	0.61 (0.38)
9R. This child does not feel comfortable outside the playground	0.67 (0.45)	0.75 (0.56)
10R. This child does not feel at ease in the group	0.80 (0.63)	0.83 (0.69)
11. This child actively seeks the company of other children	0.52 (0.27)	0.61 (0.37)
12. This child really enjoys the games and play material at the daycare center	0.67 (0.45)	0.67 (0.45)

*R, reversed for analyses. All standardized factor loadings were significant at p < 0.001. R^2^ is presented in parentheses.*

The next step was to conduct a simultaneous analysis of equal form, which means a least restricted solution. This resulted in an acceptable model fit (χ^2^(131) = 985.500, *p* < 0.00001, CFI = 0.95, TLI = 0.95, RMSEA = 0.09, SRMR = 0.05). The SRMR was good. All other fit indices were acceptable. We then restricted the factorial means by setting them to 0 for boys, which assumes non-equality. The equality constraint on the means of the factor well-being did not significantly alter the model fit, which means that boys and girls did not differ on well-being, (χ^2^(11) = 10.012, *p* > 0.05, CFI = 0.96, TLI = 0.96, RMSEA = 0.08, SRMR = 0.05).

To further establish the gender invariance, gender was used as a covariate in the MIMIC analysis. A non-significant effect of gender on well-being was found (*ß* = −0.03, *p* > 0.05), which means that boys and girls had a similar factor mean on well-being. This result confirmed findings of previous research and also our hypothesis that there is no difference between boys and girls on the LICW-D.

### Concurrent Validity

As hypothesized, we found a significant but tiny negative correlation between well-being and frustration (*r* = −0.09, *p* < 0.01) and a significant small positive correlation between well-being and soothability (*r* = 0.29, *p* < 0.001). The soothability scale was positively oriented, which explains the positive correlation. In addition, we found a significant small negative correlation with externalizing problems (*r* = −0.14, *p* < 0.001), a significant medium negative correlation with internalizing problems (*r* = −0.49, *p* < 0.001), and with total behavior problems (*r* = −0.34, *p* < 0.001). These findings confirmed our hypotheses that children who score high on well-being score low on difficult temperament and the different types of behavior problems that were measured.

## Discussion

This study examined the validity of the LICW-D in a large Norwegian ECEC toddler sample using CFA. In addition, the factorial invariance across gender and concurrent validity were examined. The study found a marginally acceptable fit for the hypothesized one-factor model. Additionally, although the fit of the modified LICW-D was slightly better for boys than girls, factorial invariance across gender was confirmed. Lastly, the modified model showed a satisfactory concurrent validity. Children with a high score on well-being scored lower on difficult temperament and internalizing, externalizing, and total behavior problems. These findings might form the starting point for further research and development of the LICW-D.

Although the first hypothesis was confirmed, the measurement errors of four item pairs were allowed to correlate to reach a marginally acceptable model fit. The definition of the modified LICW-D model as “marginally acceptable” was mainly caused by the non-acceptable RMSEA. An explanation for the high RMSEA could be that RMSEA measures absolute fit and does not have any corrections based on how simple or complex a model is. A one-factor model provides limited possibilities to find out why the RMSEA is high. However, exploring the two-factor model did not improve the RMSEA. In addition, we studied the main potential sources of misspecifications, such as the number of factors, the indicators, and the error theory in the one-factor model (Brown, 2015). None of these additional analyses provided a statistical explanation for a potential misspecification. Our findings showed that items 2, 7, and 11 had the lowest factor loadings and *R*^2^. These items were also part of the four item pairs that were allowed to correlate. Moreover, item 11 was part of two item pairs. The item pairs were often measuring the “extremes” of the same concept. For example, if the child actively seeks the company of the other children (item 11) and if the child tends to avoid contacts with other children (item 6). The involvement of these three items in the highest measurement errors might influenced the model fit as well. However, after taking these items out one by one, the model fit did not improve. Moreover, the internal consistency was still good, but with a slightly lower Cronbach’s alpha of 0.84 compared to the original 12-item LICW-D with a Cronbach’s alpha of 0.87. Considering these findings and the fact that these items did not exceed the cutoff values, we had no reason to remove them. Nevertheless, there is room for improvement of the LICW-D. Therefore, we began to reevaluate the items from a more theoretical and conceptual perspective.

First, we argue that a one-factor structure with 12 items is too simple to grasp such a complex theme as children’s well-being. There is a lengthy debate regarding the definition of well-being (Dodge et al., 2012), and different concepts are used interchangeably to describe well-being, such as quality of life and wellness (Cooke et al., 2016). Moreover, most well-being theories and measurements focus on adult well-being (Røysamb, 2014). Examination of children’s well-being asks for a different approach, however, as children are more dependent on a nurturing and supportive environment, which affects their well-being and later life outcomes (Moser et al., 2017). We therefore speculate that a more in-depth study is needed on the definition of children’s well-being to reevaluate the definition in Van IJzendoorn et al. (1998) and to examine whether more items are needed to study children’s well-being in ECEC.

Second, some of the current items might be subject to multiple interpretations. For example, item 2 focuses on whether the child does not feel at ease with some of the children. Previous research (Howes, 1987; Borge, 2014) showed that peer relations are important for children’s well-being and positive adjustment. However, even during the first years of life, children show a preference for one or two children within a larger peer group. They differentiate between available playmates and often maintain established relationships and routines with their friends (Howes, 1983, 1987). As a result, children may not interact with some of the children in the group. In addition, the established friendships are a protective factor, which means that even if a child does not feel at ease with some of the children in the group, he/she might still have a strong sense of well-being (Borge, 2014). Therefore, item 2 needs to be reevaluated.

Item 7, which focuses on whether the child has difficulty saying goodbye to the parent and is distressed or inconsolable, might not be a representation of the child’s actual level of well-being at the ECEC center. This separation situation is a complex interplay between children, parents, and professional caregivers, and children are more sensitive to what is happening when they feel vulnerable and insecure (Klein et al., 2010). It does not provide insights on children’s feelings toward a caregiver in a diversity of situations and during the day. For this reason, item 1, assessing whether the child enjoys attending the daycare setting, provides a better representation of a child’s actual well-being at the ECEC center.

Last, some of the items do not take individual and cultural differences into account. An example is item 11, assessing whether the child actively seeks the company of other children. In Norway, children’s choices and autonomy are highly valued and on the political agenda. Autonomy might promote learning motivation, self-regulation, self-control, development, and later life outcomes (Organization for Economic Co-operation and Development [OECD], 2015). Children’s well-being in ECEC in Norway is related to the opportunity to participate, to be active, and to be responsible, which also means that children have a large degree of freedom to choose their activities (Storli and Sandseter, 2019). This means that if children prefer to play alone, they are allowed to do that. Children not actively seeking the company of other children might still have a strong sense of well-being. Therefore, an item could be included assessing whether the child likes to play alone. Considering these potential limitations of items 2, 7, and 11, which had the lowest factor loadings and *R*^2^ in this study, we recommend that future studies examine the applicability of these items and the potential need for additional items.

The LICW-D correlated in the hypothesized ways with the soothability and frustration scales of the ECBQ short version, and the internalizing, externalizing, and total behavior problems scales of the CBCL-TRF/11/2-5. However, some of the scales had different correlation sizes in this study compared to previous research (De Schipper et al., 2004a,b; Gevers Deynoot-Schaub and Riksen-Walraven, 2006). In this study, the correlation with the frustration scale was tiny compared to the small correlation that was found with difficult temperament in the studies by De Schipper et al. (2003, 2004a). An explanation for our smaller correlation could be that we used a different instrument to measure difficult temperament than De Schipper et al. (2003, 2004a), who used the Infant Characteristics Questionnaire (ICQ) (Bates et al., 1979). Even though both instruments were developed to measure difficult temperament, it could be that the scales have a slightly different focus. It seems that the frustration scale of the ECBQ short version focuses on negative affect related to interruption of ongoing tasks or goal blocking, whereas the ICQ focuses mainly on difficulty to adapt to novelty, in addition to irritable distress. Another explanation might be that in our study, professional caregivers rated children’s difficult temperament, whereas in De Schipper et al. (2003, 2004a), mothers rated children’s difficult temperament. However, the soothability scale showed a small and similar correlation compared to scale that was used by De Schipper et al. (2003, 2004a). Nevertheless, in addition to previous research, the present study showed tiny to small correlations between difficult temperament and children’s low level of well-being, even though difficult temperament was measured using different scales and by different raters than in previous research.

Moreover, both previous research and this study confirmed that children’s behavior problems are correlated with children’s low level of well-being, even though there was a difference in the correlation size on the internalizing scale and small incongruence between some studies on the externalizing behavior problems scale. The higher correlation for internalizing behavior problems than for externalizing behavior problems suggests that internalizing problems are a better indication of low well-being. Moreover, these results show that a distinction can be made between children’s low level of well-being and the different types of problem behaviors.

A strength of this study is the large sample that allowed for rigorous testing of the LICW-D within a CFA framework. However, there are some limitations worth mentioning. One limitation is that children’s well-being is not measured directly, as we could not use children as respondents. This is a common limitation when studying young children. Knowing something about how the children view their well-being in addition to the correlations found would have offered valuable information about the concurrent validity of the LICW-D. Given the young age of the children, we were dependent on the ratings by professional caregivers, however. We recommend that future studies with older children in ECEC include children’s perspectives as well.

Another limitation is that we did not have information on the professional caregivers that filled out the questionnaires for the children. Professional caregivers’ characteristics may have possibly influenced the way they see the child and as a result might have affected their responses. Therefore, future research should include professional caregivers’ characteristics to study the potential effect of respondents’ characteristics.

Even though there are some limitations, our findings show that the LICW-D has the potential to become a well-validated instrument to map the level of well-being for children in ECEC. However, as our findings demonstrate, some adaptations might be needed. Therefore, future research should study the LICW-D in other countries as well to examine cross-cultural validity. In addition, we recommend reexamination of the definition of children’s well-being, followed by extension of the LICW-D to include extra items to study children’s well-being in ECEC even more accurately. Moreover, the applicability of items 2, 7, and 11 needs to be reconsidered. Further development of the LICW-D might form the base for a well-validated tool that can be used by national and international researchers, policy makers, and practitioners to measure children’s well-being in ECEC.

## Data Availability Statement

The datasets presented in this article are not readily available because the data analyzed in this study is subject to the following licenses/restrictions: We are not allowed to share data outside the key personnel for the grant by the Norwegian Centre for Research Data (NSD). Requests to access the datasets should be directed to ES: elisabet.solheim@r-bup.no.

## Ethics Statement

The studies involving human participants were reviewed and approved by the Regional Committees for Medical and Health Research Ethics South East Norway (REK 2017/430) and by the Norwegian Centre for Research Data (NSD 332636) and is registered at clinicaltrials.gov, identifier NCT03879733. Written informed consent to participate in this study was provided by the participants’ legal guardian/next of kin.

## Author Contributions

All authors listed have made a substantial, direct, and intellectual contribution to the work, and approved it for publication.

## Conflict of Interest

The authors declare that the research was conducted in the absence of any commercial or financial relationships that could be construed as a potential conflict of interest.

## Publisher’s Note

All claims expressed in this article are solely those of the authors and do not necessarily represent those of their affiliated organizations, or those of the publisher, the editors and the reviewers. Any product that may be evaluated in this article, or claim that may be made by its manufacturer, is not guaranteed or endorsed by the publisher.

## References

[B1] Achenbach T. M. Rescorla L. A. (2000). *Manual for the ASEBA Preschool Forms and Profiles.* Burlington, VT: University of Vermont, Research Center for Children, Youth and Families.

[B2] Bates J. E. Freeland C. A. B. Lounsbury M. L. (1979). Measurement of infant difficultness. *Child Dev.* 50:3. 10.2307/1128946 498854

[B3] Ben-Arieh A. (2008). The child indicators movement: Past. Present, and Future. *Child Indic. R.* 1:1. 10.1007/s12187-007-9003-1

[B4] Blakemore S.-J. Frith U. (2005). *The Learning Brain: Lessons for Education.* Oxford: Blackwell Publishing. 10.1111/j.1467-7687.2005.00434.x 16246234

[B5] Borge A. I. H. (2014). “Psychology of child well-being: measuring well-being,” in *Handbook of Child Well-Being. Theories, Methods and Policies in Global Perspective*, eds Ben-Arieh A. Casas F. Frønes I. Korbin J. E. (Dordrecht: Springer), 597–599.

[B6] Bradshaw J. Richardson D. (2009). An index of child well-being in Europe. *Child Indic. R.* 2:3. 10.1007/s12187-009-9037-7

[B7] Brown T. A. (2015). *Confirmatory Factor Analysis for Applied Research*, 2nd Edn. New York, NY: Guilford Press.

[B8] Comrey A. L. Lee H. B. (1992). *A First Course in Factor Analysis*, 2nd Edn. Hillsdale: NJ: LawrenceErlbaum Associates.

[B9] Cooke P. J. Melchert T. P. Connor K. (2016). Measuring well-being: a review of instruments. *Couns. Psychol.* 44:5. 10.1177/0011000016633507

[B10] Council of the European Union (2019). *Council Recommendation on High-Quality Early Childhood Education and Care Systems.* Brussels: Council of the European Union.

[B11] De Schipper J. C. Tavecchio L. W. C. Van IJzendoorn M. H. Linting M. (2003). The relation of flexible child care to quality of center day care and children’s socio-emotional functioning: a survey and observational study. *Inf. Beh. Dev.* 26:3. 10.1016/S0163-6383(03)00033-X

[B12] De Schipper J. C. Tavecchio L. W. C. Van IJzendoorn M. H. Van Zeijl J. (2004a). Goodness-of-Fit in center day care: relations of temperament, stability, and quality of care with the child’s adjustment. *Early Child. R. Quart.* 19:2. 10.1016/j.ecresq.2004.04.004

[B13] De Schipper J. C. Van IJzendoorn M. H. Tavecchio L. W. C. (2004b). Stability in center day care: relations with children’s well-being and problem behavior in day care. *Soc. Dev.* 13:4. 10.1111/j.1467-9507.2004.00282.x

[B14] Department for Education and Child Development (2016). *Wellbeing for Learning and Life: A Framework for Building Resilience and Wellbeing in Children and Young People.* Hindmarsh: Department for Education and Child Development, Government of South Australia.

[B15] Dodge R. Daly A. P. Huyton J. Sanders L. D. (2012). The challenge of defining wellbeing. *J. Wellb.* 2:3. 10.5502/ijw.v2i3.4 32285354

[B16] Fabrigar L. R. Wegener D. T. (2012). *Exploratory Factor Analysis.* New York, NY: Oxford University Press. 10.1093/acprof:osobl/9780199734177.001.0001

[B17] Field A. (2018). *Discovering Statistics Using IBM SPSS Statistics*, 5th Edn. London: SAGE.

[B18] Flora D. B. Curran P. J. (2004). An empirical evaluation of alternative methods of estimation for confirmatory factor analysis with ordinal data. *Psychol. Meth.* 9:4. 10.1037/1082-989X.9.4.466 15598100 PMC3153362

[B19] Gevers Deynoot-Schaub M. Riksen-Walraven J. M. (2006). Peer interaction in child care centres at 15 and 23 months: stability and links with children’s socio-emotional adjustment. *Inf. Beh. Dev.* 29:2. 10.1016/j.infbeh.2005.12.005 17138283

[B20] Howes C. (1983). Patterns of friendship. *Child Dev.* 54:4. 10.2307/1129908

[B21] Howes C. (1987). Social competence with peers in young children: developmental sequences. *Dev. Rev.* 7:3. 10.1016/0273-2297(87)90014-1

[B22] Hu L.-T. Bentler P. M. (1999). Cutoff criteria for fit indexes in covariance structure analysis: conventional criteria versus new alternatives. *Stru. Eq. Mod.: Multid. J.* 6:1. 10.1080/10705519909540118

[B23] Huppert F. A. (2009). Psychological well-being: evidence regarding its causes and consequences. *App. Psychol. Health Wellb.* 1:2. 10.1111/j.1758-0854.2009.01008.x

[B24] Huppert F. A. So T. T. (2013). Flourishing across europe: application of a new conceptual framework for defining well-being. *Soc. Indic. R.* 110:3. 10.1007/s11205-011-9966-7 23329863 PMC3545194

[B25] Jung E. Yoon M. (2016). Comparisons of three empirical methods for partial factorial invariance: forward, backward, and factor-ratio tests. *Struct. Equ. Model.* 23:4. 10.1080/10705511.2015.1138092

[B26] Klein P. S. Kraft R. R. Shohet C. (2010). Behaviour patterns in daily mother-child separations: possible opportunities for stress reduction. *Early Child Dev. Care* 180:3. 10.1080/03004430801943290

[B27] Kline R. B. (2016). *Principles and Practice of Structural Equation Modeling*, 4th Edn. New York, NY: Guilford Press.

[B28] Lekhal R. Drugli M. B. Berg-Nielsen T. S. Solheim Buøen E. (2020). A model of intervention and implementation of quality building and quality control in childcare centers to strengthen the mental health and development of 1-3-year olds: protocol for a randomized controlled trial of Thrive by Three. *JMIR Res. Protoc.* 9:e17726. 10.2196/17726 32773366 PMC7592061

[B29] Lyubomirsky S. Sheldon K. M. Schkade D. (2005). Pursuing happiness: the architecture of sustainable change. *Rev. Gen. Psychol.* 9:2. 10.1037/1089-2680.9.2.111

[B30] MacCallum R. C. Browne M. W. Sugawara H. M. (1996). Power analysis and determination of sample size for covariance structure modeling. *Psychol. Meth.* 1:2. 10.1037/1082-989X.1.2.130

[B31] Mashford-Scott A. Church A. Tayler C. (2012). Seeking children’s perspectives on their wellbeing in early childhood settings. *Int. J. Early Child* 44:3. 10.1007/s13158-012-0069-7

[B32] Moser T. Broekhuizen M. L. Leseman P. P. M. Melhuish E. (2017). *Theoretical Framework: A Brief Integration of Literature Reviews by ISOTIS Work Packages. ISOTIS, Inclusive Education and Social Support to Tackle Inequalities in Society.* Available online at: http://www.isotis.org/wp-content/uploads/2017/05/D2.1_Report20170428.pdf (Accessed August 23, 2021)

[B33] Muthén L. K. Muthén B. O. (2017). *Mplus. Version 8 (computer software).* Los Angeles, CA: Muthén and Muthén.

[B34] National Scientific Council on the Developing Child (2007). *A Science-Based Framework forEarly Childhood Policy: Using Evidence to Improve Outcomes in Learning, Behavior, and Health for Vulnerable Children.* Cambridge, MA: Center on the Developing Child, Harvard University.

[B35] Nussbeck F. W. Eid M. Lischetzke T. (2006). Analysing multitrait-multimethod data with structural equation models for ordinal variables applying the WLSMV estimator: what sample size is needed for valid results? *Br. J. Math. Stat. Psychol.* 59:1. 10.1348/000711005X67490 16709286

[B36] Organization for Economic Co-operation and Development [OECD] (2009). *Doing Better for Children.* Paris: OECD.

[B37] Organization for Economic Co-operation and Development [OECD] (2012). *Starting Strong III. A Quality Toolbox for Early Childhood Education and Care.* Paris: OECD.

[B38] Organization for Economic Co-operation and Development [OECD] (2015). *Early Childhood Education and Care. Policy Review Norway.* Paris: OECD.

[B39] Putnam S. P. Jacobs J. F. Gartstein M. A. Rothbart M. K. (2010). *Development and Assessment of Short and Very Short Forms of the Early Childhood Behavior Questionnaire.* Poster presented at International Conference on Infant Studies. Baltimore, MD.

[B40] Putnick D. L. Bornstein M. H. (2016). Measurement invariance conventions and reporting: the state of the art and future directions for psychological research. *Dev. R.* 41 71–90. 10.1016/j.dr.2016.06.004 27942093 PMC5145197

[B41] Røysamb E. (2014). “Psychology of child well-being: measuring well-being,” in *Handbook of Child Well-Being: Theories, Methods and Policies in Global Perspective*, eds Ben-Arieh A. Casas F. Frønes I. Korbin J. E. (Dordrecht: Springer), 568–569. 10.1007/978-90-481-9063-8

[B42] Schmitt N. Golubovich J. Leong F. T. L. (2011). Impact of measurement invariance on construct correlations, mean differences, and relations with external correlates: an illustrative example using big five and RIASEC measures. *Assessment* 18:4. 10.1177/1073191110373223 20622198

[B43] Statistics Norway (2021). *Kindergartens, 2020, Final Figures (data set).* Oslo: Statistics Norway. 10.1787/84425dd1-en

[B44] Storli R. Sandseter E. B. H. (2019). Children’s play, well-being and involvement: how children play indoors and outdoors in norwegian early childhood education and care institutions. *Int. J. Play* 8:1. 10.1080/21594937.2019.1580338

[B45] Sylva K. Ereky-Stevens K. Aricescu A.-M. (2015). *Overview of European ECEC Curricula and Curriculum Template. CARE, Curriculum and Quality Analysis and Impact Review of European Early Childhood Education and Care.* Available online at: https://ecec-care.org/fileadmin/careproject/Publications/reports/CARE_WP2_D2_1_European_ECEC_Curricula_and_Curriculum_Template.pdf (Accessed August 23, 2021).

[B46] Van IJzendoorn M. H. Tavecchio L. W. C. Stams G.-J. Verhoeven M. Reiling R. (1998). Attunement between parents and professional caregivers: a comparison of childrearing attitudes in different child-care settings. *J. Marr. Family* 60:3. 10.2307/353545

[B47] Zachrisson H. D. Lekhal R. (2014). “Psychology of child well-being: early childhood education and care,” in *Handbook of Child Well-Being: Theories, Methods and Policies in Global Perspective*, eds Ben-Arieh A. Casas F. Frønes I. Korbin J. E. (Dordrecht: Springer), 599–601.

